# Magnetic Resonance Features of a Recent Catheter Ablation of Left Posterior Fascicular Ventricular Tachycardia

**DOI:** 10.7759/cureus.16555

**Published:** 2021-07-22

**Authors:** Basel Abdelazeem, Taylor A Revere, Sarah Ayad, Saed Alnaimat, Mustafa Hassan

**Affiliations:** 1 Internal Medicine, McLaren Health Care, Flint/Michigan State University, Flint, USA; 2 Medical Education, Michigan State University College of Human Medicine, Flint, USA; 3 Internal Medicine, Rutgers New Jersey Medical School/Trinitas Regional Medical Center, Elizabeth, USA; 4 Cardiology, McLaren Health Care, Flint/Michigan State University, Flint, USA

**Keywords:** case report, ventricular tachycardia, catheter ablation, magnetic resonance imaging, electrocardiogram

## Abstract

A 71-year-old male patient with a past medical history of hypertension, hyperlipidemia, and chronic kidney disease stage II presented with a complaint of intermittent palpitations for three months and was found to have wide complex tachycardia on the electrocardiogram (ECG). The patient was given adenosine and amiodarone, following which he underwent synchronized cardioversion at 150 Joules followed by 200 Joules without successful conversion. He was subsequently initiated on lidocaine drip at the rate of 1 to 4 mg/minute to maintain adequate rhythm control, which converted him to sinus rhythm and relieved his symptoms. An eventual assessment with an electrophysiology study identified the presence of incessant left ventricular tachycardia (VT). The mechanism was confirmed to be left posterior fascicular ventricular tachycardia (LPF-VT). Successful mapping and ablation for the LPF-VT were achieved. Post-procedure cardiac MRI showed two small areas of near-transmural delayed enhancement. These areas are associated with nulled areas in the inferolateral wall at the left posterior His-Purkinje fascicle. This case highlights fascicular VT as a separate clinical entity, with its characteristic ECG features and acute MRI features after ablation.

## Introduction

Left posterior fascicular ventricular tachycardia (LPF-VT) is a rare form of idiopathic ventricular tachycardia (VT) believed to originate from a reentrant circuit located in the Purkinje network. Most often, this tachycardia originates from the left posterior or anterior fascicle [1]. It can often be mixed up with supraventricular tachycardia (SVT) with the right bundle branch block (RBBB) and left anterior fascicular block (LAF-B). Here, we present a case of LPF-VT and highlight how to identify electrocardiogram (ECG) features and acute MRI features of LPF-VT after ablation.

## Case presentation

A 71-year-old male presented with complaints of intermittent palpations for three months with increasing frequency and new onset of lightheadedness for two days. His past medical history was pertinent for hypertension for more than 35 years, hyperlipidemia, and chronic kidney disease stage II for more than 20 years. Home medications included losartan 50 mg and metoprolol 25 mg every day.

**Figure 1:**
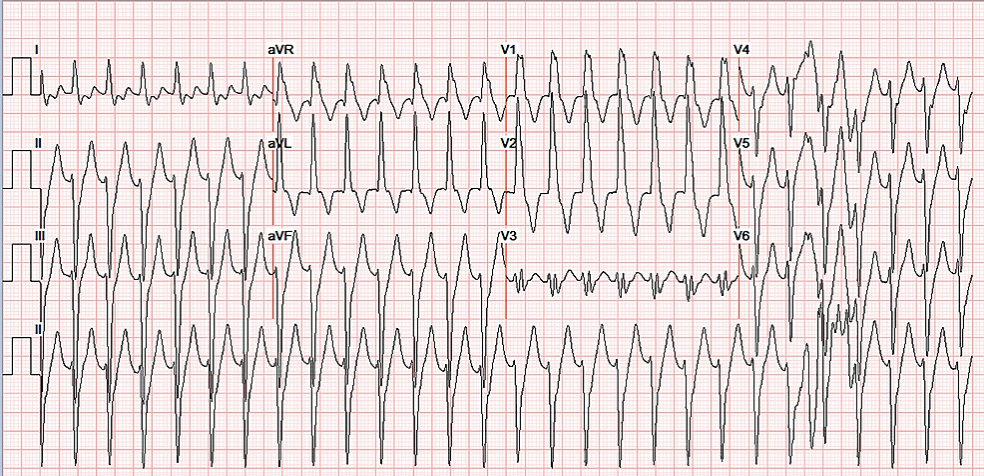
ECG. ECG performed in ED showing relatively narrow complex tachycardia at a heart rate of 164 beats per minute and QRS duration of 149 ms. Rsr’ morphology in V1 is consistent with VT rather than RBBB. ECG also demonstrates the LAF-B. ECG: electrocardiogram; ED: Emergency Department; LAF-B: left anterior fascicular block; RBBB: right bundle branch block; VT: ventricular tachycardia

On arrival to the emergency department, vital signs showed a blood pressure of 127/97 mmHg, respiratory rate of 18 breaths per minute, temperature of 98°F, saturation of 97% on room air, and tachycardia with a heart rate of 141 beats per minute. The cardiac examination showed tachycardia, regular rhythm, with no murmurs, rubs, or gallop. Peripheral pulses were 2+ and equal bilaterally. Laboratory workup, including complete blood count, comprehensive metabolic panel, and thyroid-stimulating hormone, was within normal limits. Serial troponin-I was negative. Brain natriuretic peptide was elevated at 483 pg/mL. A chest X-ray showed no acute cardiopulmonary process. An ECG performed in the Emergency Room showed wide complex tachycardia at a rate of 164 beats per minute and QRS duration of 149 ms, with a LAF-B (Figure 1). He received 150 amiodarone bolus and was placed on an amiodarone drip without change in the heart rate or rhythm. Synchronized cardioversion was attempted at 150 Joules followed by 200 Joules, which was not successful.

**Figure 2:**
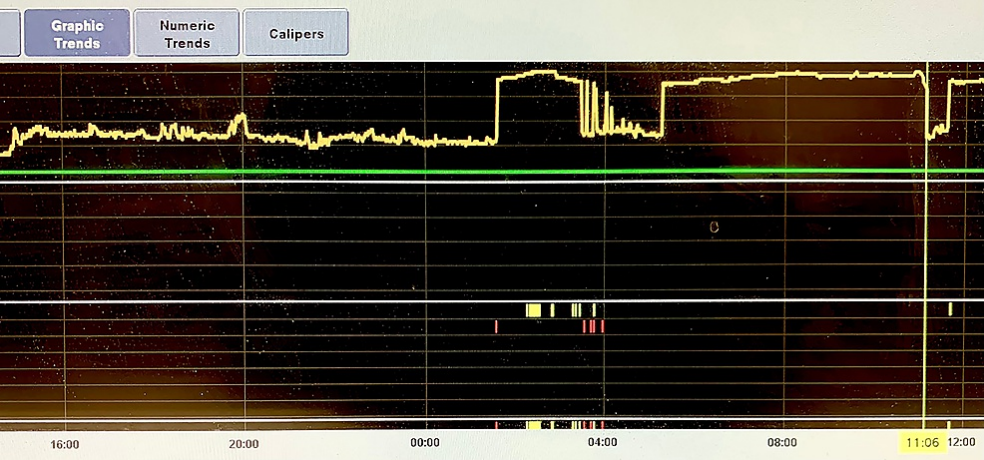
Inpatient telemetry showing a sudden-onset–sudden-offset pattern of fascicular VT. VT: ventricular tachycardia

**Figure 3:**
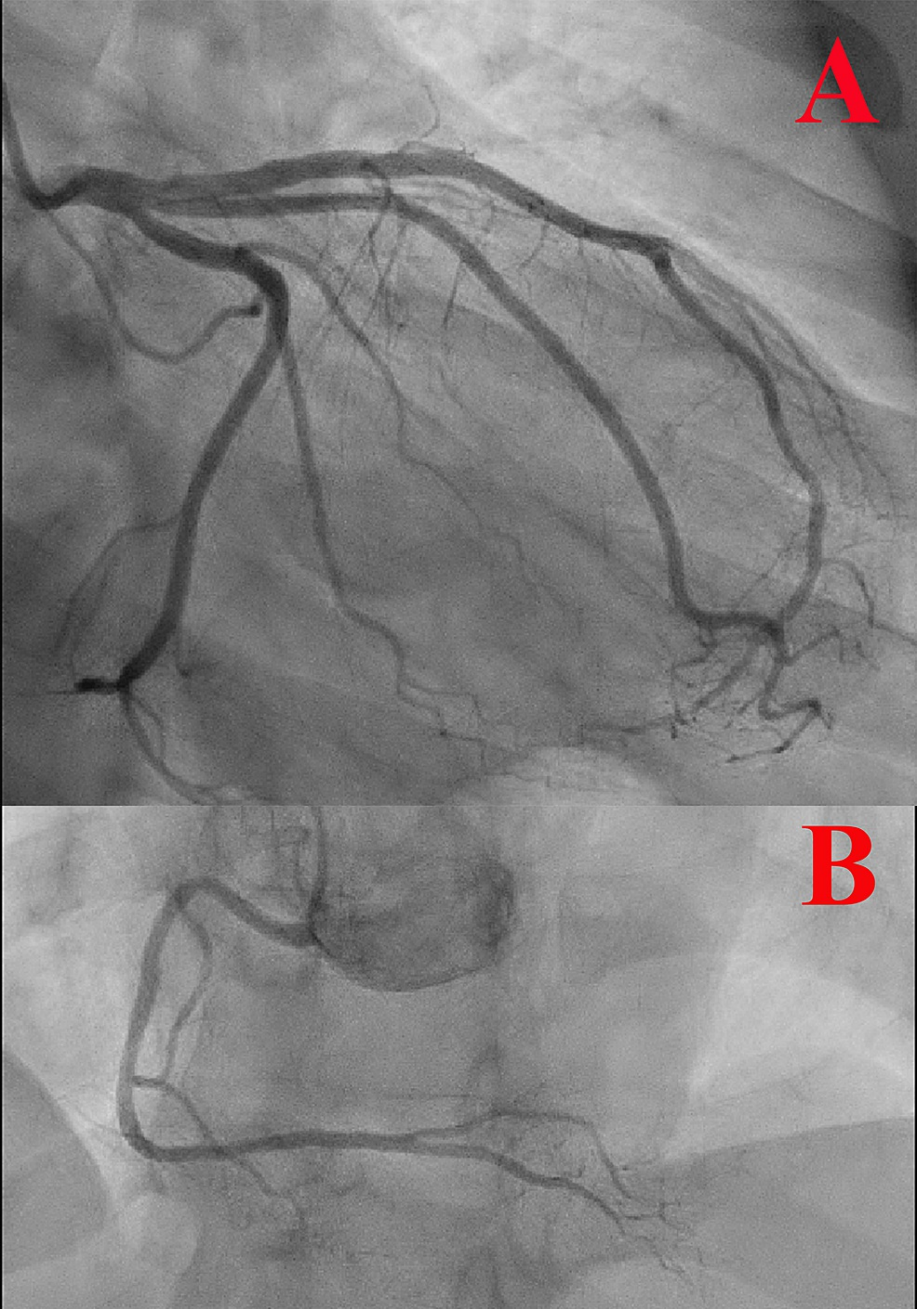
Coronary angiogram. (A) Selective left coronary angiogram showing normal left coronary circulation. (B) Selective right coronary angiogram showing normal right coronary circulation.

**Figure 4:**
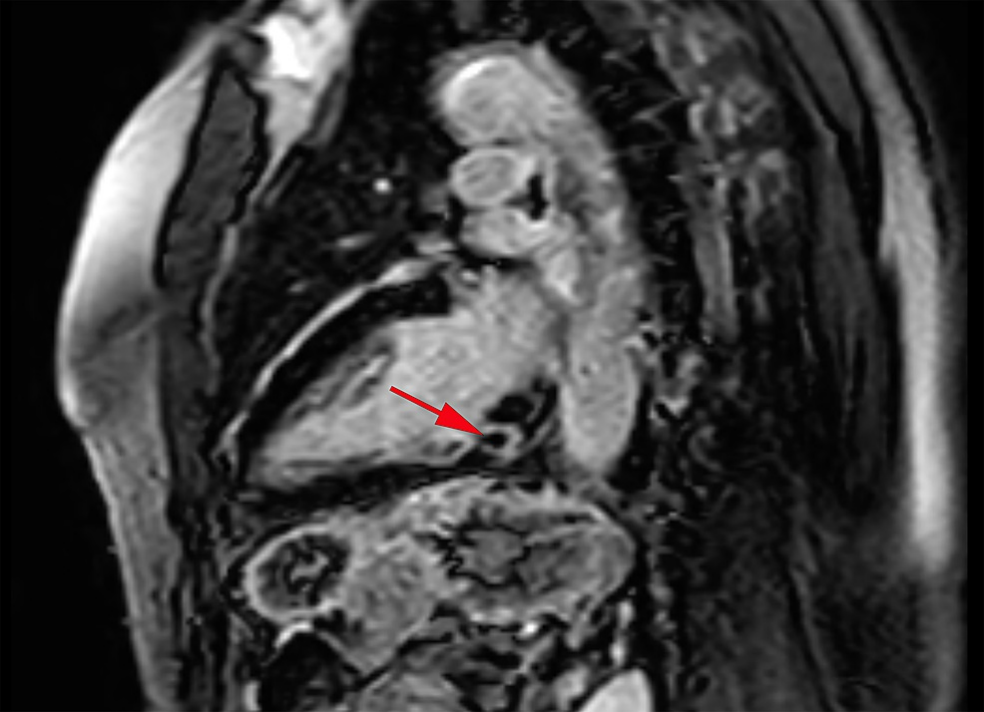
Cardiac MRI. Cardiac MRI showing near-transmural delayed enhancement and nulled area within the delayed enhancement in the basal inferior wall due to a recent ablation procedure. Red arrow points to the nulled area. MRI: magnetic resonance imaging

Afterward, the patient was started on lidocaine 100 mg bolus and drip at a rate of 1 to 4 mg/minute to maintain adequate rhythm control, with subsequent conversion to normal sinus rhythm. During hospitalization, the patient had frequent recurrences of small runs of VT which were terminated with verapamil and lidocaine (Figure 2). The ECG demonstrated a left ventricular ejection fraction of 50-55% and mild tricuspid and pulmonary valve regurgitation. The coronary angiogram showed normal coronary arteries (Figure 3). Although cardiac MRI was considered prior to ablation, it could not be done due to the unavailability of the in-house cardiac MRI. Therefore, the patient had an outpatient cardiac MRI a few days later that demonstrated normal-sized right and left ventricles. Left ventricular systolic function and rest perfusion were found to be normal with an ejection fraction of 72%. There was near-transmural delayed enhancement with nulled areas within the delayed enhancement. The right ventricular function was mildly reduced, but no abnormal enhancement within the right ventricular myocardium was noted. The valves appeared normal in structure. The visualized aorta, pulmonary artery, and main branches also appeared normal (Figure 4).

**Figure 5:**
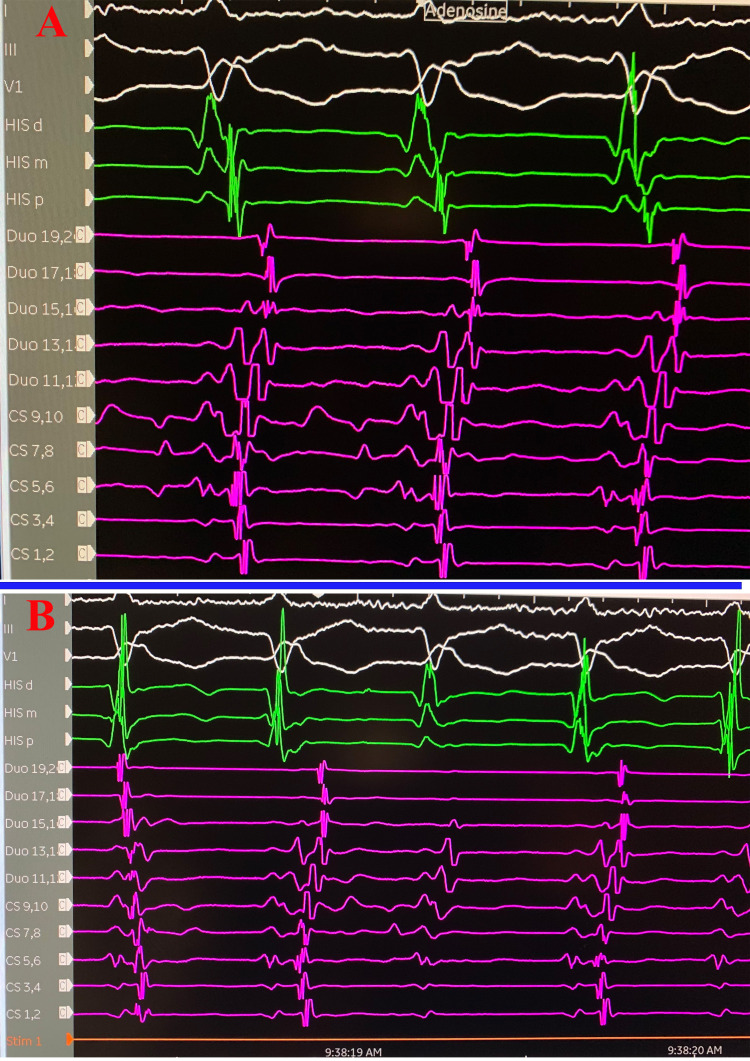
Adenosine challenge during EP. Adenosine challenge during EP study confirming a ventricular origin rather than a supraventricular origin of the tachycardia. (A) Before giving adenosine. (B) After giving adenosine. EP: electrophysiology

Our patient’s initial ECG revealed wide complex tachycardia at a heart rate of 164 beats per minute and QRS duration of 149 ms with LAF-B. Rsr’ morphology in V1 was consistent with VT rather than RBBB (Figure 1). The patient underwent an electrophysiology (EP) study and ablation. During the EP study, 12-lead ECG was similar to previously found tachycardia with RBBB morphology, left axis deviation, and relatively narrow QRS. The tachycardia cycle length was about 400 ms with a 1:1 atrioventricular (AV) relationship. Retrograde atrial activation was noted with a concentric atrial activation sequence. He received adenosine 6 mg with no effect. Subsequently, 12 mg of adenosine resulted in a retrograde block of tachycardia in the atrium with a continuation of tachycardia without interruption resulting in AV dissociation without any effect in tachycardia (Figure 5). This maneuver confirmed the mechanism as a fascicular left ventricular tachycardia mechanism originating from the left posterior His-Purkinje fascicle. Successful mapping and ablation for the LPF-VT were executed. VT was noninducible after the ablation despite aggressive stimulation protocol and isoproterenol infusion of up to 10 µg/minute.

The patient tolerated the procedure well. He was discharged on aspirin, losartan, atorvastatin, and verapamil. He also received a LifeVest and was monitored with outpatient follow-up.

## Discussion

VT is a tachycardia of ventricular origin lasting more than three beats. It is classified as nonsustained if it lasts less than 30 seconds or sustained if it lasts more than 30 seconds in duration. In a patient without structural heart disease, idiopathic VT originates from the right ventricular outflow tract, left ventricular outflow tract, or left ventricular fascicular systems (posterior, anterior, upper septal) [2]. The most common form of idiopathic VT that originates from the fascicular systems is LPF-VT [3].

LPF-VT is typically idiopathic, occurring in patients between the ages of 15 and 40, and is more common in males than females. LPF-VT is commonly confused with SVT with RBBB and LAF-B [4]. A study by Michowitz et al. proposed four parameters that can be utilized to support the diagnosis of a left posterior VT: atypical RBBB-like V1 morphology (no rsR’ or R larger than R’ in lead V1), a QRS width of ≤140 ms, a positive QRS in lead aVR, and V6 R/S ratio of ≤1. On the other hand, ECG features that support SVT with RBBB or LAF-B aberrancy include a more typical V1 morphology, QRS width >140 ms, negative QRS in lead aVR, and V6 R/S ratio of >1 [4].

Cardiac MRI was recommended to evaluate for any underlying cardiac abnormality that may predispose to fascicular tachycardia [2]. A cardiac MRI was done a few days after the ablation procedure, revealing normal right ventricle, valves, aorta, and pulmonary arteries. The left ventricle ejection fraction was 72%. The ablation site was seen as a near-transmural delayed enhancement with nulled areas within delayed enhancement and was interpreted as consistent with possible subacute myocardial infarction in the basal inferolateral wall in the right coronary artery or obtuse marginal territory. Interestingly, the patient had normal coronary arteries on the coronary angiogram, and transmural delated enhancement seen on cardiac MRI was solely due to the ablation procedure.

Management of fascicular tachycardia includes pharmacological therapy and ablation. Verapamil is the drug of choice for acute treatment; unfortunately, it is not effective as a chronic treatment [5]. Catheter ablation is the mainstay of treatment with a success rate of 70-90%. Recurrence rates after successful ablation range from 0 to 20% [4,5].

## Conclusions

LPF-VT is the most common type of VT and originates from a reentrant circuit located in the Purkinje network. ECG features of LPF-VT include QRS width less than 120-140 ms, RBBB, and left axis deviation. We presented the case of a 71-year-old male patient who presented with palpitations and was diagnosed with LPF-VT to highlight ECG features and acute MRI features of LPF-VT after ablation. Management of this condition includes pharmacological and catheter ablation. Successful ablation requires correct identification of the source of abnormal rhythm.
